# The Effect of Wealth Shocks on Loss Aversion: Behavior and Neural Correlates

**DOI:** 10.3389/fnins.2017.00237

**Published:** 2017-04-27

**Authors:** V. S. Chandrasekhar Pammi, Sergio Ruiz, Sangkyun Lee, Charles N. Noussair, Ranganatha Sitaram

**Affiliations:** ^1^Centre of Behavioural and Cognitive Sciences, University of AllahabadAllahabad, India; ^2^Laboratory for Brain-Machine Interfaces and Neuromodulation, Pontificia Universidad Católica de ChileSantiago, Chile; ^3^Department of Psychiatry, Faculty of Medicine, Interdisciplinary Center for Neuroscience, Pontificia Universidad Católica de ChileSantiago, Chile; ^4^Department of Neurology, Baylor College of MedicineHouston, TX, USA; ^5^Economic Science Laboratory, Eller College of Management, University of ArizonaTucson, AZ, USA; ^6^Institute for Biological and Medical Engineering, Pontificia Universidad Católica de ChileSantiago, Chile

**Keywords:** neural loss aversion, wealth shocks, value function, ventral prefrontal cortex, loss aversion, agency

## Abstract

Kahneman and Tversky (1979) first demonstrated that when individuals decide whether or not to accept a gamble, potential losses receive more weight than possible gains in the decision. This phenomenon is referred to as loss aversion. We investigated how loss aversion in risky financial decisions is influenced by sudden changes to wealth, employing both behavioral and neurobiological measures. We implemented an fMRI experimental paradigm, based on that employed by Tom et al. (2007). There are two treatments, called RANDOM and CONTINGENT. In RANDOM, the baseline setting, the changes to wealth, referred to as wealth shocks in economics, are independent of the actual choices participants make. Under CONTINGENT, we induce the belief that the changes in income are a consequence of subjects' own decisions. The magnitudes and sequence of the shocks to wealth are identical between the CONTINGENT and RANDOM treatments. We investigated whether more loss aversion existed in one treatment than another. The behavioral results showed significantly greater loss aversion in CONTINGENT compared to RANDOM after a negative wealth shock. No differences were observed in the response to positive shocks. The fMRI results revealed a neural loss aversion network, comprising the bilateral striatum, amygdala and dorsal anterior cingulate cortex that was common to the CONTINGENT and RANDOM tasks. However, the ventral prefrontal cortex, primary somatosensory cortex and superior occipital cortex, showed greater activation in response to a negative change in wealth due to individual's own decisions than when the change was exogenous. These results indicate that striatum activation correlates with loss aversion independently of the source of the shock, and that the ventral prefrontal cortex (vPFC) codes the experimental manipulation of agency in one's actions influencing loss aversion.

## Introduction

It is common for individuals to experience unanticipated changes to wealth. In economics, these are referred to as wealth *shocks*. One might suddenly incur unexpected medical expenses, traffic fines, new tax liabilities, changes in monthly utility bills or a decline in the value of one's investments. Similarly, one might receive a windfall, a positive wealth shock, in the form of an unanticipated pay increase, a holiday bonus, or an appreciation in the value of an investment. Such wealth shocks may be small or large and can potentially influence financial decision-making through a variety of channels. These include a change in the willingness to take risks due to the shift in wealth itself, an updating of beliefs about future income uncertainty, a shift of the reference level of wealth, or a change in the emotional state of the agent when a decision is made (Kimball, 1990; Terzi et al., 2016).

In this paper we study financial decisions in response to sudden anticipated shocks to wealth. We measure both behavioral and neurobiological responses to a shock. We conduct an experiment, in which each individual decides sequentially whether or not to accept 256 gambles, using a paradigm adapted from that employed by Tom et al. (2007). Subjects begin with an initial endowment of 20 Euro. During the task, we subject participants' endowments to shocks in the following manner. After 64 trials, participants experience an unanticipated wealth shock of −10 Euro, and after 64 more trials there is a positive shock of 20 Euro.

The principal research question we consider is whether the source of a wealth shock influences how much the shock affects subsequently estimated levels of loss aversion, the unwillingness to accept a risk that may result in a loss. Experimental research indicates that the majority of individuals are risk averse (Binswanger, 1980; Holt and Laury, 2002; Harrison et al., 2012). However, prior studies disagree about whether individuals become more likely to accept a given gamble if their wealth experiences a shock.

Moreover, in addition to the change in income that the wealth shock induces, it may also affect an agent's belief about the overall level of risk she faces. An individual is temperate (Kimball, 1992) if she responds to an increase in background risk by making more risk-averse choices. Most recent work finds that a majority of individuals are temperate (Ebert and Wiesen, 2014; Noussair et al., 2014). If temperate individuals respond to unanticipated wealth shocks by believing that income uncertainty is greater, they would become less prone to take risks after the shock, regardless of whether the shock increases or decreases their wealth.

It is also possible that a shock changes the reference level of wealth an individual uses in evaluating her choices. Prospect theory (Kahneman and Tversky, 1979), a behaviorally-inspired theoretical model of decision making, postulates a discrete change in the slope and the curvature of the utility function at a reference monetary value. Empirical estimates indicate that the average individual is roughly indifferent between receiving her reference payoff with certainty and playing a lottery in which individuals have a 50% chance of losing an amount x or gaining 2 x relative to her reference point (Tversky and Fox, 1995; Tom et al., 2007). This can be interpreted as indicating that losses have twice the weight as gains, so that the loss aversion coefficient is equal to two. If the reference point is not fully updated in response to a shock (see for example Köszegi and Rabin, 2007), then the estimated loss aversion coefficients would change after each shock.

In addition to the economic parameters discussed above, there is also reason to believe that the emotional state of an individual might influence how the shocks affect his willingness to accept risks. It is known that emotional state can affect decision-making under risk (Hsee and Rottenstreich, 2004; De Martino et al., 2006; Knutson et al., 2008; Nguyen and Noussair, 2014; Breaban et al., 2016). The emotional state may differ depending on how the wealth shock arose.

In our experiment, we manipulate the source of the wealth shock. In addition to the baseline treatment described above, called RANDOM, we conducted a second treatment, called CONTINGENT. In CONTINGENT, we induce the belief that the changes in income are a result of the choices of the participants. Actual changes in endowment are the same in CONTINGENT and RANDOM, allowing behavioral and neural measures to be directly compared between treatments. We focus on how behavior and brain activation depend on the source of the wealth shock.

We are interested in whether more loss aversion exists in one treatment than another. We compare the estimated loss aversion coefficient from the choice data between the two treatments, after both a negative and a positive wealth shock. We also compare the level of brain activation in the ventral prefrontal cortex (vPFC) and the ventral striatum (VS), the two brain regions associated with loss aversion in previous studies (Tom et al., 2007; Brooks et al., 2010; Pammi et al., 2015).

## Methods

### Participants

Seventeen participants underwent two fMRI scanning sessions, and performed two experimental tasks, called CONTINGENT and RANDOM (see Supplementary Material for Instructions to the participants). Though the same participants took part in both tasks, the instructions emphasized the different source of the wealth shocks. The time interval between the tasks varied from 1.5 h to 15 days. The order of CONTINGENT and RANDOM for this study was not counterbalanced, with CONTINGENT preceding RANDOM. Two participants' data was removed due to inconsistent behavioral responses and scanner related issues. The remaining 15 participants' data was analyzed. The participants were five male and ten female students at the University of Tübingen, aged 18–30 (average age = 26.6 years). They were recruited for the experiment with a paper advertisement. All participants were right-handed, with no present or previous history of psychiatric/neurological disorders (Wittchen et al., 1997), no medical condition affecting the central nervous system, and no substance dependence or abuse. All participants had scores greater than 28/30 points on the mini-mental state examination (Folstein et al., 1975). Written informed consent was obtained from each participant, and approval for the research was obtained from the ethics committee of the Faculty of Medicine, University of Tübingen, Germany. Participants were informed that a minimum of 5 Euros and a maximum of 60 Euros per task would be paid for their participation in both experimental tasks.

### Behavioral parameters

In each of the experimental tasks, CONTINGENT and RANDOM, participants' decisions (to accept or reject a gamble) and their decision times (time from onset of stimulus till the decision was made) for each trial were recorded. Based on a participant's 256 decisions, we computed a behavioral loss aversion coefficient (LAC) for each participant. These behavioral parameters were analyzed using a repeated measures ANOVA.

### fMRI parameters

A standard EPI sequence 3T TIM Trio whole-body scanner (Siemens, Erlangen, Germany), with the following parameters was used: TR = 1,500 ms, TE = 30 ms, number slices = 16. A T1-weighted structural scan was collected from each participant using the following pulse sequence parameters: MPRAGE, matrix size = 256 × 256, 160 partitions, 1 mm^3^ isotropic voxels, TR = 2,300 ms, TE = 3.93 ms, TI = 1100 ms, α = 8°. The above protocols were repeated in the four experimental runs, which constituted each of the two tasks. The functional images were analyzed using General Linear Model of SPM8.

### Experimental procedures

While supine in the fMRI scanner, each participant performed the two experimental tasks (CONTINGENT and RANDOM) in two separate experimental sessions. The CONTINGENT task was conducted first and then followed by the RANDOM task. Each task, CONTINGENT and RANDOM, consisted of 4 runs. Each run consisted of 64 trials of 5 s each, presented in immediate succession. The 5 s duration of each trial consisted of a 4-s decision phase followed by a 1- s fixation cross (inter-trial gap).

Participants were given 20 Euro as initial endowment at the beginning of each experimental task. In each trial, a 50/50 gamble was presented, representing a 50% chance of a monetary loss and a 50% chance of a gain. For example, one trial consisted of a 50% chance of losing 19 Euro and a 50% chance of gaining 38 Euro, relative to the initial endowment of Euro 20 (Euro 1 equaled approximately US$1.20 at the time the experiment was conducted). The payoffs in the gambles were chosen to include every element in an 8 × 8 lossxgain matrix, as in Tom et al. (2007) and Pammi et al. (2015). Loss values ranged from 5 to 20, with a step size of 2, and gain values ranged from 10 to 40, with a step size of 4 (values are in terms of Euros).

**Figure 1:**
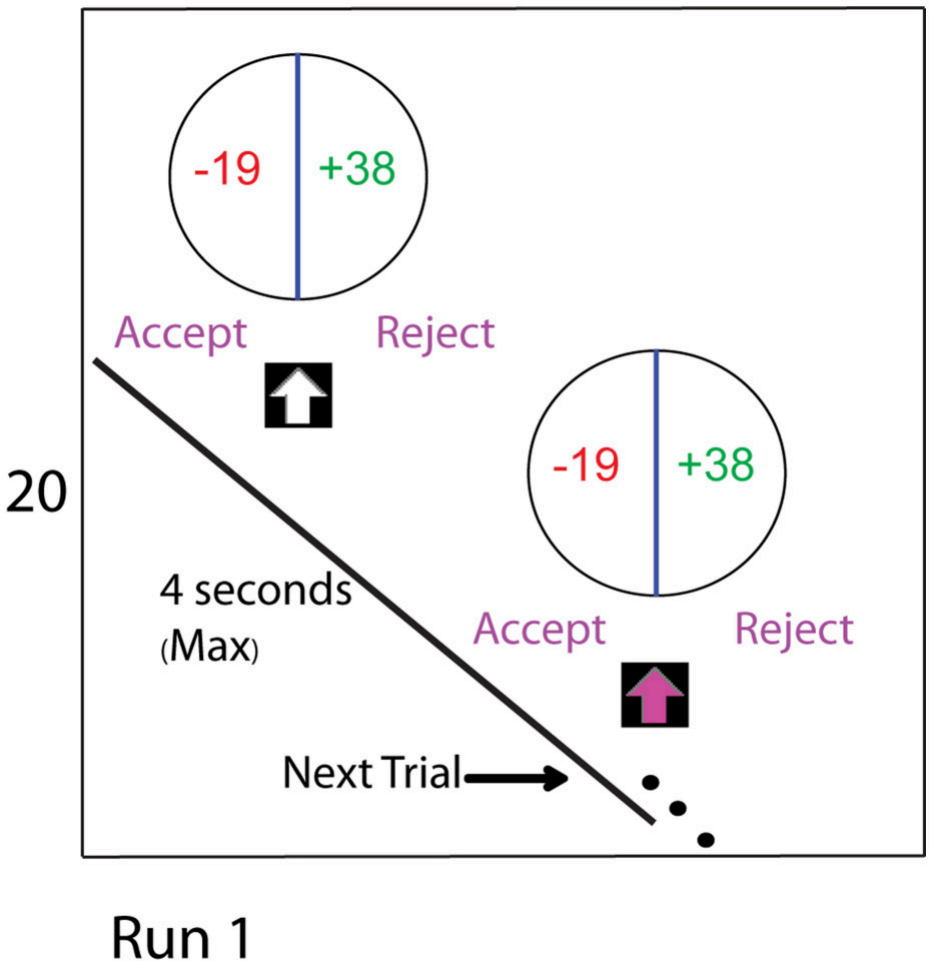
**The experimental task consists of four runs**. The two manipulations comprising this study were the CONTINGENT and the RANDOM treatments with endowment values specified at the beginning of each run. The figure shows the display seen during a trial of Run 1. In the CONTINGENT task, the current wealth for each run was obtained from decisions in the previous run. In the RANDOM task, the current wealth was set by the experimenter at the beginning of each run. In both of the tasks, participants begin with an initial endowment of 20 Euro in Run 1, which was displayed on the left side of the screen as shown in the figure for the entire duration of the run. During the task, we subject their endowment to shocks. After 64 trials they experience a wealth shock of −10 Euro, and after 64 more trials there is a positive shock of 20 Euro. In our baseline setting (RANDOM), the changes in endowment are independent of the actual choices that subjects make. In CONTINGENT, we induce the belief that the changes in income are a result of consequential choices of the participants. Actual Changes in endowment are the same in CONTINGENT and RANDOM, allowing behavioral and neural measures to be directly compared between treatments.

Participants were told that each trial was independent of the other trials. Their task was to accept or reject the gamble presented in each trial. Accepting a gamble would yield participants either the loss or gain amounts specified in that trial. In the example trial shown in Figure 1, the loss and gain values are −19 and +38, respectively, from the initial endowment. Rejecting the gamble would result in the initial endowment of Euro 20. Participants were required to make their choice within 3 s after the arrow turned to pink. A fixation-cross against the black screen acted as an inter-trial gap. Participants were instructed to start thinking about their choice to accept or reject from the onset of the stimulus, but could confirm their choice only after the arrow turned to pink. If they took longer than 3 s to make their decision, the stimulus presentation program proceeded to the next trial after recording that there was no decision.

In the CONTINGENT task, the initial endowment was Euro 20 for the first run and the wealth available for subsequent runs was obtained from the outcome of previous runs. In this task, participants were instructed that at the end of each run, six trials would be randomly chosen to count toward their payoff. They would receive the average payoff of these six trials. Therefore, participants were aware that the average payoff at the end of each run could yield a better or worse payoff than their wealth at the beginning of the run. They were instructed that at the end of each run, verbal feedback about the success or failure of the gamble would be provided, and that the amount remaining after the resolution of the gambles would be carried over to subsequent runs.

We determined the amounts for all participants in the same manner, and those amounts were added or subtracted from participants' cash balance at the end of each run. Participants in the CONTINGENT task started with Euro 20 as an initial endowment. At the end of the first run they were made to lose Euro 10, leaving them with Euro 10 for the second run. At the end of second run they were made to win 20 Euros, leaving them with Euro 30 for the third run. From the third run to fourth run, they were made to retain the same amount, Euro 30.

In the RANDOM task, participants were instructed that they would perform trials in each run with different initial balances of wealth at the beginning of each run and that these wealth levels would be fixed by the experimenter. The starting values for each run were 20, 10, 30, and 30 Euros, representing therefore a decrease, increase, and maintenance of wealth from one run to the next. Importantly, although the computation of gains and losses was performed at the end of each run, participants were informed that no feedback would be provided about the success or failure of their gambles after each run, and rather that feedback would be provided only at the end of the fourth run.

To make both tasks comparable (Figure 1), we manipulated the changes in the endowment value for both the tasks (CONTINGENT and RANDOM) in such a way that the same values (i.e., 20, 10, 30, 30 Euros) for each run were used. Participants were also informed that a minimum of 5 Euros and a maximum of 60 Euros per task would be paid for their participation in the experiment. However, for ethical reasons, at the end of the experiment, and irrespective of their performance, the maximum amount of money was paid to each participant. The experimental design software package, COGENT 2000 (developed by the Functional Imaging Laboratory, University College London), was used for the experimental design and data acquisition.

### Behavioral analysis

Behavioral loss aversion coefficients were computed by conducting ordinary least squares regressions on each individual decision. A participant's decision to accept or reject a gamble, and the magnitudes of the gain and the loss were the dependent variables. Dividing the beta coefficient on losses by that on the gains yielded a loss aversion coefficient (LAC). The LAC was estimated for each run and each individual separately. To compute the coefficients, we ran OLS regressions with the following specification:

$$\begin{array}{l} {\text{Y}}_{\text{it}}=\beta_{0}+\beta_{1}^{*}{\text{Loss}}_{\text{t}}+\beta_{2}^{*}{\text{Gain}}_{\text{t}} \end{array}$$

where Y_it_ = 1 if subject i chooses the risky option in trial t, Loss_t_ is the possible loss if the gamble is chosen at time t, and Gain_t_ is the possible gain if the gamble is chosen at time t. The regression is estimated separately for each participant i. The ratio of the coefficients “−β_1_/β_2_” is taken as the Loss Aversion Coefficient (LAC) for the individual. We also used the pooled data for all subjects to estimate an aggregate LAC. This single model yielded β_1_ and β_2_ values and based on which the loss aversion coefficients was computed for each run. The estimated loss aversion coefficients for a representative individual were 2.06, 2.54, 2.55, and 2.75 respectively, in the four runs of CONTINGENT. They were 2.58, 2.63, 2.63, and 2.76 in the four runs in RANDOM.

The loss x gain matrix of 8 × 8 (as in Tom et al., 2007) was reduced to 4 × 4 for the behavioral analysis. The decision times (DT) for each run were also recorded. The analyses of LAC and DT aimed at quantifying differences, between the CONTINGENT and RANDOM tasks, in the behavioral response to the wealth changes during each task. To obtain wealth-change-related effects, we computed differences in these measures between consecutive runs. The Run2-1, Run3-2, Run4-3 differences for both behavioral parameters, namely LAC and DT, were computed as Run2 values subtracted from Run1, Run3 values subtracted from Run2, and Run4 values subtracted from Run3, respectively. Factorial models were constructed as 2 factors (task: CONTINGENT and RANDOM) x 3 factors (consecutive run differences: Run2-1, Run3-2, Run4-3) for both LAC and DT.

### FMRI analysis

Functional images were analyzed using SPM8 (Wellcome Department of Imaging Neuroscience, University College London) utilizing standard first and second level models. The functional imaging data were pre-processed using standard procedures such as realignment and unwarping, spatial normalization, and smoothing (with an 8 mm Gaussian kernel). In the first level analysis, the decision phase was modeled using decision times measured from the stimulus onset to the confirmation of response. Thus, modeling the decision phase with decision times allowed us to use for naturally occurring jitter. Separate first level models were constructed for CONTINGENT and RANDOM for every participant. Because CONTINGENT and RANDOM were performed in different scanning sessions, we modeled them separately. We then constructed a Random effects second level model pooling the data from both tasks. Each first-level design matrix was constructed using functional images from all four runs for decision phase duration. In both the brain activation and the behavioral data analysis the onset and offset of each trial is an event regressor, and the gain and loss magnitudes of the lottery available in that trial is a parametric modulator. The dependent variable, the choice to accept or reject, was obtained from the reduced payoff matrix of size 4 × 4 (Tom et al., 2007). The parametric modulation procedure allowed us to dissociate the brain activations related to potential loss and gain magnitudes. The model also included motion parameters as regressors for each run.

Separate models were constructed for each of the two tasks. This allowed us to identify the brain regions whose activations correlated with neural loss aversion in each task separately. This also permitted us to identify activation patterns that correlate with loss aversion, independently of the origin of the wealth shock. Following Dreher (2007) and Tom et al. (2007), we obtained brain regions that responded parametrically with losses and gains for each task. A two (task: CONTINGENT and RANDOM) × four (Neural LACs: Run1, Run2, Run3 and Run4) factorial design at the second level (Glascher and Gitelman, 2008) was constructed by pooling neural loss aversion related contrast images from each first level model. This factorial model resulted in brain activations pertaining to loss aversion systems for the two tasks and modulations across the four runs. In order to find a common or shared network of brain regions subserving both tasks and both positive and negative wealth shocks, we constructed a contrast by equally weighting all eight conditions in the factorial model. In order to obtain brain activations due to a task (CONTINGENT vs. RANDOM), we constructed differential statistical contrasts between tasks for each of the four runs.

Neural loss aversion (Dreher, 2007; Tom et al., 2007) was computed in the first-level models for the brain regions responding with decreasing activity in the loss modulator and increased activity in the gain modulator. The neural loss aversion was calculated for each of the two tasks separately. The neural loss aversion at every voxel was obtained by subtracting the parameter estimate (slope) of the gain response from that of the loss response (negative slope). The mean beta values corresponding to the neural loss aversion as reported in **Figures 3**, **4** and Table 1 were extracted over a spherical volume of 8 mm ROI from the 2 × 4 factorial model and these values for used for the computation of correlations and quantification of each of the tasks.

**Table 1:** **Neural loss aversion differences between task for each run: CONTINGENT > RANDOM**.

**Brain area**		**BA**	**Coordinates (mm)**			**T Score (Number of voxels in the cluster)**
Run 1 (CONTINGENT > RANDOM) No activations						
Run 2 (CONTINGENT > RANDOM)						
Primary somatosensory cortex/Postcentral	R	3	24	−36	50	5.37 (534)
Ventral prefrontal cortex/Rostral anterior cingulate cortex	R	47/32	24	34	10	4.26 (315)
Ventral prefrontal cortex/Rostral anterior cingulate cortex	R	24/32	18	20	28	4.67 (315)
Supplementary motor area/Middle cingulate cortex	R	6/4	12	−18	48	4.42 (326)
Superior occipital cortex	R	19/18	18	−82	18	4.22 (318)
Run 3 (CONTINGENT > RANDOM) No activations						
Run 4 (CONTINGENT > RANDOM) No activations						

*Stereotaxic MNI coordinates of significant BOLD signals with cluster level FWE corrected p < 0.05 and the statistical threshold of peak voxels of the cluster T > 4.22*.

In addition, we also calculated correlations between the neural-loss-aversion-related brain activations (ROIs reported in Table 1) and the behavioral loss aversion coefficients (LAC) for each run and condition separately, with each individual as the unit of observation. In addition, correlations were performed for the areas observed common in both the tasks (**Figure 3**). The mean beta values of the General Linear Model (GLM) were extracted over a spherical volume of 8 mm from the 2 × 4 factorial model and these values were used for the computation of correlations and quantification of each of the tasks. This results are reported in **Figure 5**.

## Results

### Behavioral results

The behavioral loss aversion coefficients (LAC) and decision times (DT) were analyzed using a 2 factor (Experimental task: CONTINGENT and RANDOM) × 3 factor (Main effect of run: Run 2-1, Run 3-2, Run 4-3) repeated measures ANOVA.

#### Behavioral loss aversion coefficient (LAC)

**Figure 2:**
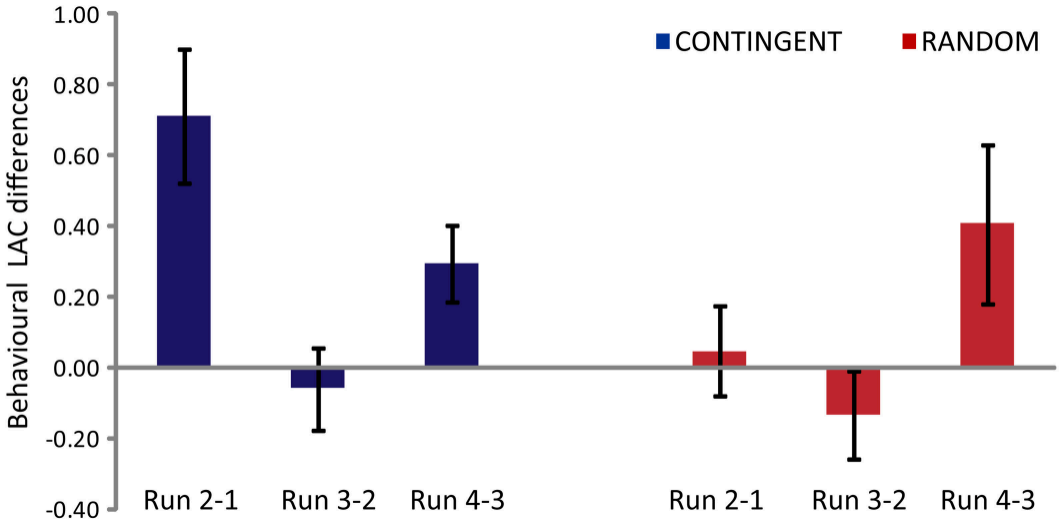
**Behavioral Loss Aversion Coefficient (LAC) differences between one run and the next**. Y-axis represents differences in the behavioral loss aversion coefficient values for CONTINGENT and RANDOM tasks.

The main effect of the experimental task [*F*_(1, 14)_ = 6.169, *p* = 0.026] and the main effect of run [*F*_(2, 28)_ = 5.328, *p* = 0.011] were found to be significant (Figure 2). The interaction (experimental task x main effect of run) was also found to be significant [*F*_(2, 28)_ = 5.833, *p* = 0.03]. The *post-hoc* analysis (Duncan correction) revealed that theRun2-1 difference was significantly greater in CONTINGENT than in RANDOM, indicating a greater increase in loss aversion in CONTINGENT than in RANDOM. In CONTINGENT, Run3-2 is significantly smaller (*p* < 0.05) than Run2-1. However, in the RANDOM task, the LACs in Run2-1 and Run3-2 were not significantly different from each other. This shows that the average LAC in the CONTINGENT task was higher than in RANDOM. Furthermore, the difference between Run2-1 and Run3-2 in CONTINGENT is significant. The average coefficients with standard error across participants' were 2.17 ± 0.24, 2.88 ± 0.30, 2.82 ± 0.27, and 3.11 ± 0.32 respectively, in the four runs of CONTINGENT. They were 3.01 ± 0.33, 3.05 ± 0.36, 2.92 ± 0.27, and 3.33 ± 0.44 in the four runs of RANDOM. Thus, the loss aversion increased more in response to the negative shock in the CONTINGENT than the RANDOM task.

#### Decision time (DT)

The difference between tasks (CONTINGENT and RANDOM) was non-significant [*F*_(1, 14)_ = 2.115, *p* = 0.168]. However, Main effect of run [*F*_(2, 28)_ = 6.327, *p* = 0.005] were found to be significant. The result indicates that the CONTINGENT and RANDOM tasks took a similar amount of time and thus were presumably similarly demanding. The average decision times (in seconds) and their standard errors are 1.61 ± 0.06, 1.49 ± 0.04, 1.46 ± 0.04, and 1.47 ± 0.04 respectively, in the four runs of CONTINGENT. They were 1.44 ± 0.05, 1.40 ± 0.03, 1.41 ± 0.04, and 1.39 ± 0.03 in the four runs in RANDOM.

### Imaging results

**Figure 3:**
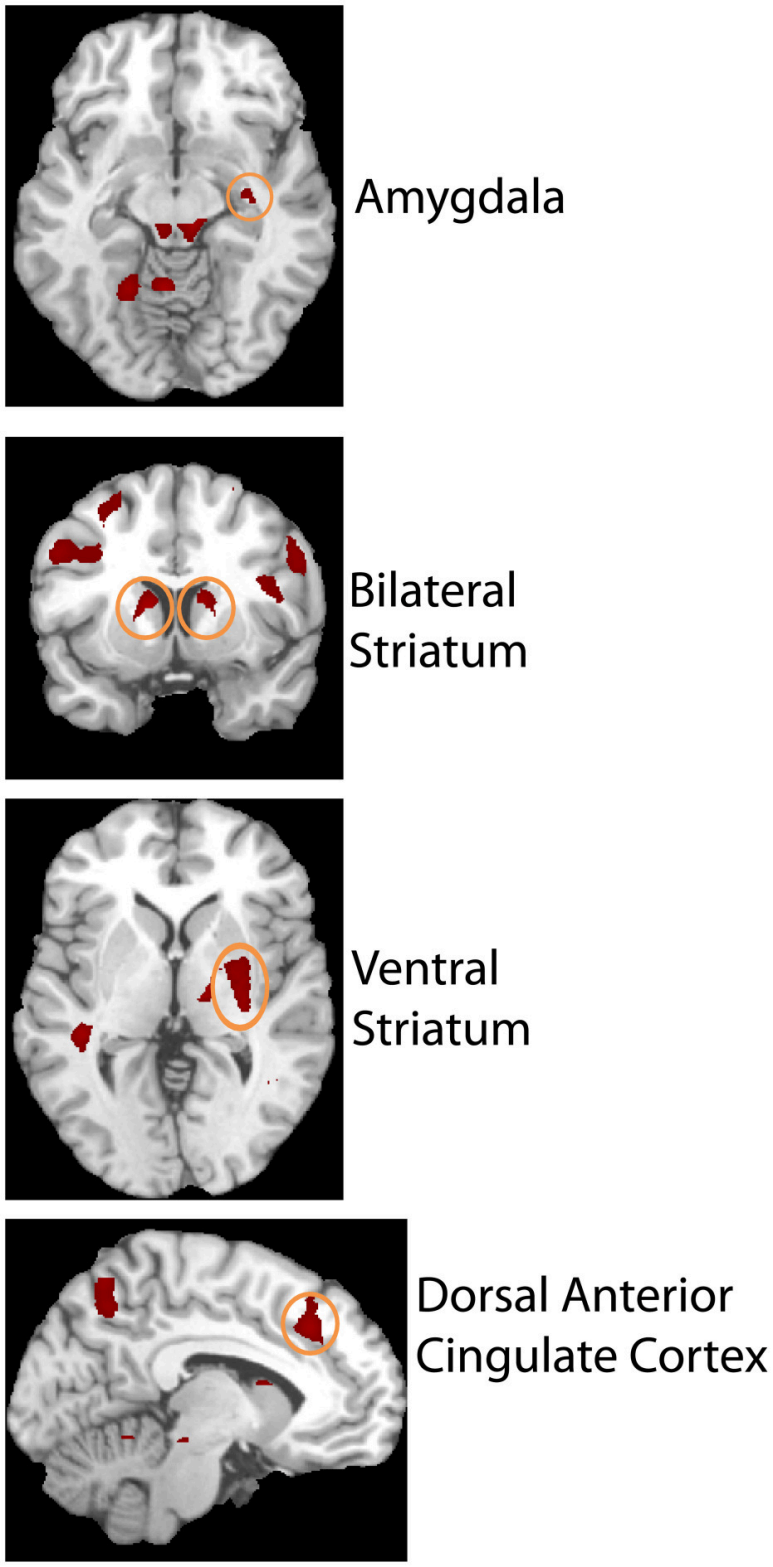
**Brain areas showing Neural Loss Aversion in both the CONTINGENT and RANDOM tasks**. Activity in right amygdala, bilateral striatum, right ventral striatum, and dorsal anterior cingulate cortex is shown.

**Figure 4:**
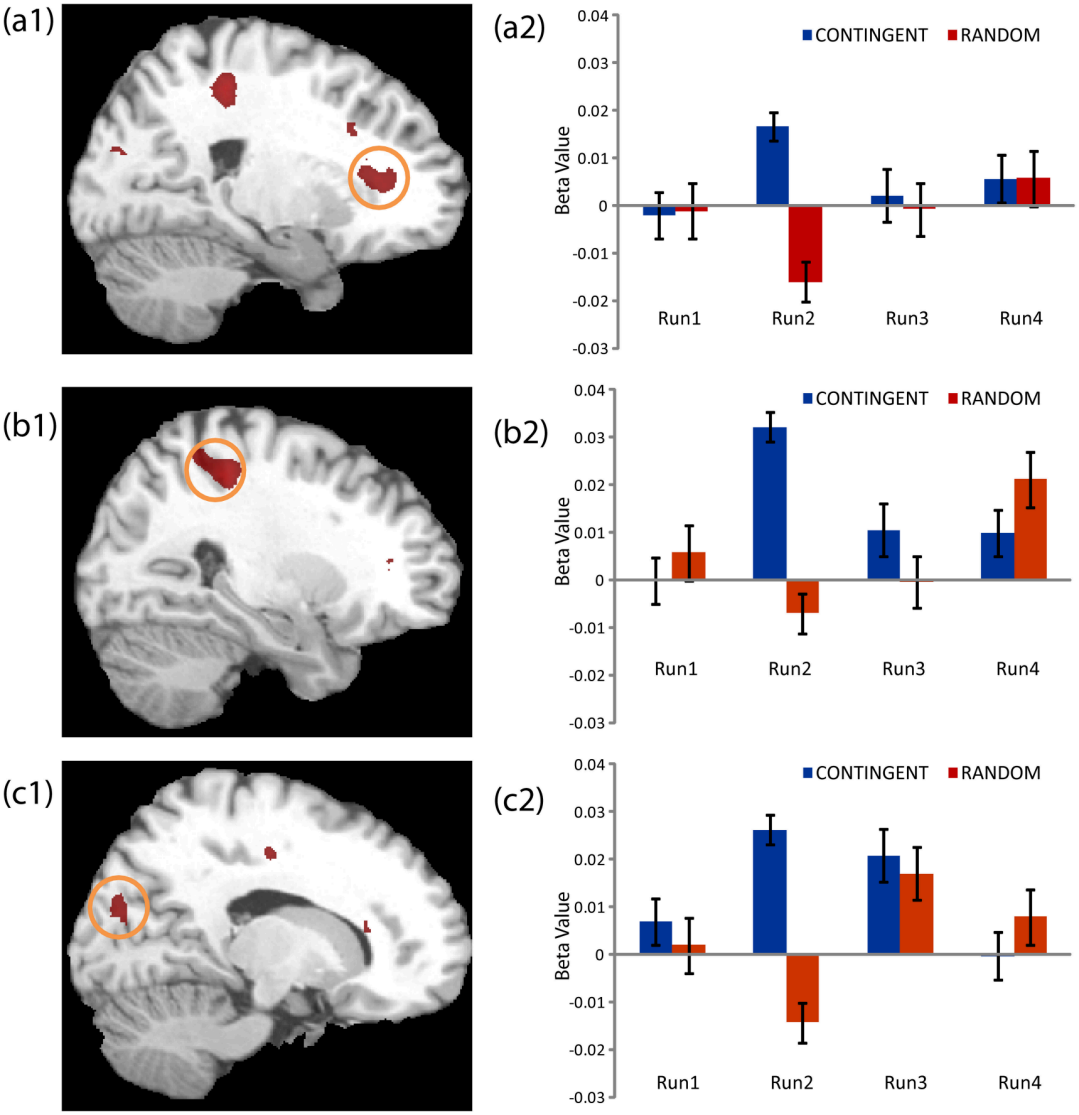
**Neural Loss Aversion Differences between CONTINGENT and RANDOM tasks**. The activity across the four runs in both tasks for the brain regions presented in Table 1 are shown. The areas illustrated arethe Ventral Prefrontal Cortex/Rostral Anterior Cingulate Cortex **(a1, a2)**, right Primary Somatosensory Cortex/Postcentral **(b1, b2)** and right Superior Occipital Cortex **(c1, c2)**.

The second level 2 × 4 factorial design yielded common and differential neural systems underlying the two tasks, as well as the loss, gain or no-change conditions present in the four runs. The common activations for neural loss aversion were found in the right amygdala, bilateral striatum, right ventral striatum and dorsal anterior cingulate cortex (Figure 3). This common network can be interpreted as describing the neural substrate underlying loss aversion. In line with the behavioral results, differential activations between the CONTINGENT and RANDOM treatments were found only for Run2 (Table 1 with threshold *T* > 4.22). The brain activations in the ventral prefrontal cortex/rostral anterior cingulate cortex (BA 47/32), right primary somatosensory cortex (S1)/postcentral (BA 3) and right superior occipital cortex (BA 19/18) are shown in Figure 4 and Figure S1. These brain activations also survived the small volume correction and hence corrected for multiple comparisons. The brain activations and the activity profiles across runs and tasks reveal the engagement of these areas in run 2 under CONTINGENT, after the negative wealth shock perceived to be a consequence of the subject's own decisions.

**Figure 5:**
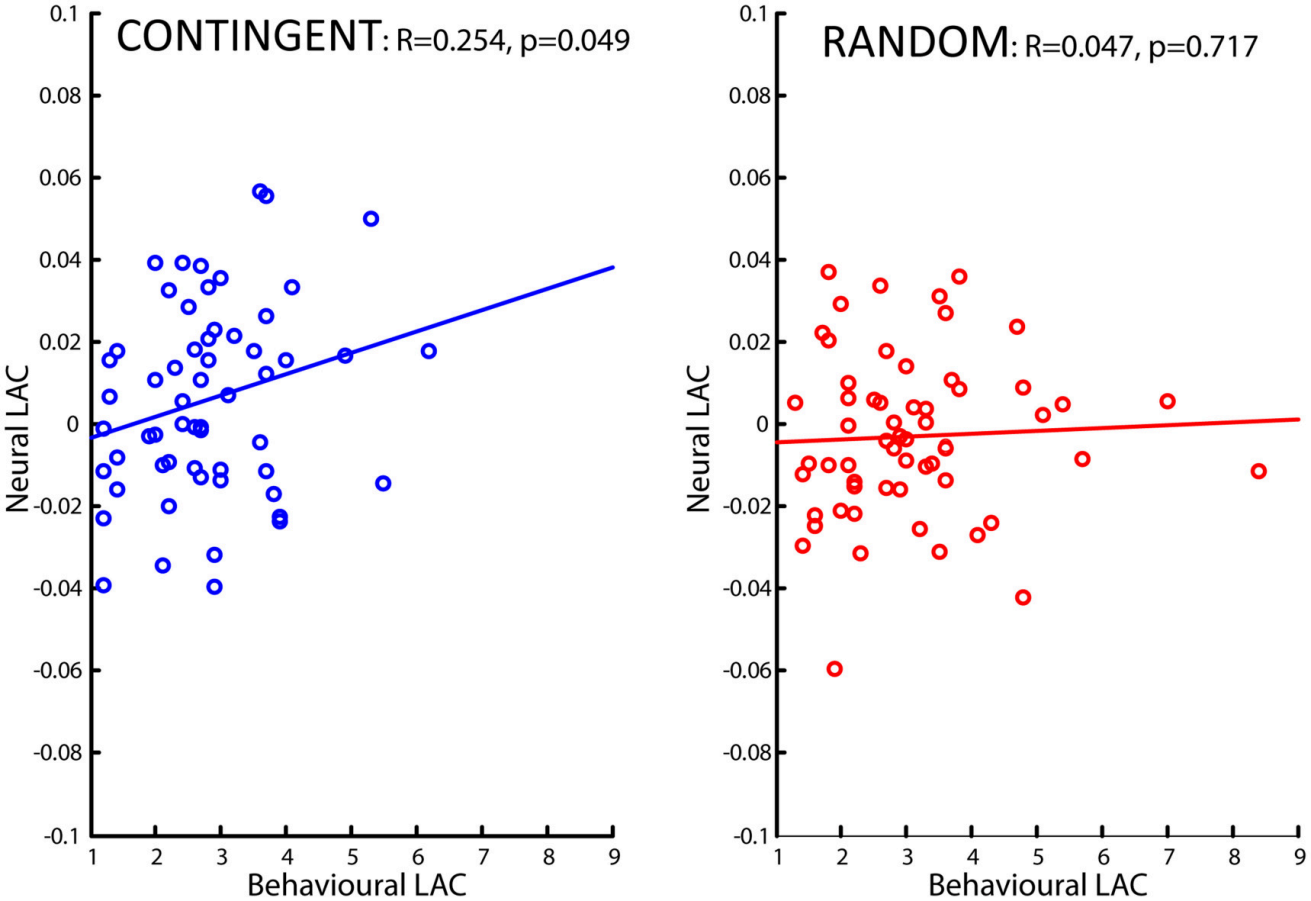
**Brain-Behavior Correlations (BBC) of the Ventral Prefrontal Cortex/Rostral Anterior Cingulate Cortex**. The BBCs of ROIs for the CONTINGENT **(Left)** and RANDOM tasks **(Right)** show significant correlations for CONTINGENT (*R* = 0.254, *p* = 0.049), in contrast to RANDOM (*R* = 0.047, *p* = 0.717). The x-axis represents Behavioral LAC values and the y-axis the Neural LAC values in the Ventral Prefrontal Cortex/Rostral Anterior Cingulate Cortex for all four runs (15 participants' data in each task).

We also performed a brain-behavior correlational analysis (BBC) between the ROIs, consisting of the loss aversion related brain areas reported in Figures 3, 4 (right amygdala, right striatum, bilateral striatum, right ventral striatum and dorsal anterior cingulate cortex, ventral prefrontal cortex/rostral anterior cingulate cortex, right primary somatosensory cortex/postcentral and right superior occipital cortex), and the behavioral loss aversion coefficients (LAC) for the pooled data for the four runs. The BBCs revealed significant correlations only in the ventral prefrontal cortex/rostral anterior cingulate cortex (peak activations reported in the Figures 3, 4 and Table 1). The BBCs (Figure 5) are significant in CONTINGENT (*R* = 0.254, *p* = 0.049) though not in RANDOM (*R* = 0.047, *p* = 0.717). This result reveals the role of the ventral prefrontal cortex/rostral anterior cingulate cortex (BA 47/32), specific to the CONTINGENT task.

## Discussion

The aim of this investigation was to find behavioral and neural correlates underlying the effect of changes in current wealth on risky choice behavior. Our experiment consisted of two different tasks, called RANDOM and CONTINGENT. In both settings, subjects choose whether to accept lotteries that either earn or lose them money. The two settings differ in that in RANDOM there were shocks to a subject's wealth over the course of the experimental session that were independent of the subjects' decisions. In CONTINGENT, subjects experience identical shocks to wealth as in RANDOM, but believe that the shocks are a consequence of their own decisions. Actual changes in wealth are the same in CONTINGENT and RANDOM, allowing behavioral and neural measures to be directly compared between treatments, controlling for current wealth and previous history. We investigated loss aversion (Abdellaoui et al., 2007; Mukherjee et al., 2017) on both the behavioral and neural levels (Kahneman and Tversky, 1979), and searched for both shared and distinct systems underlying loss aversion.

The behavioral results indicated that loss aversion was significantly greater after a negative shock under CONTINGENT than under RANDOM. This shows that the influence of a negative wealth shock on loss aversion is greater when the shock is believed to have occurred as a consequence of one's own decisions. The results from decision times showed that the two tasks took the same amount of time to perform on average.

Neurobiologically, we found a shared neural system underlying neural loss aversion, irrespective of treatment (De Martino et al., 2006, 2010; Tom et al., 2007; Brooks et al., 2010; Charpentier et al., 2015; Pammi et al., 2015), in sub-cortical areas such as the amygdala and the striatum. Furthermore, we observed a distinct cortical neural system whose activation in response to wealth shocks were different when the subject believes that the wealth shocks were due to her own choices rather than outside influences. This system is in the ventral prefrontal cortex where affect-related processes involved in decision-making have been proposed (Bechara et al., 1999). Thus, our results implicate shared neural substrates that activate with both CONTINGENT and RANDOM, as well as distinct regions that activate specifically in CONTINGENT. This result is similar to those observed for context dependence (Engelmann and Hein, 2013) or reference dependence (Brooks et al., 2010). The evidence for the role of ventral prefrontal cortex (vPFC) is consistent with its role in the processing of loss signals (Baste et al., 2010). Previous studies also show loss related effects and involvement of the ventral prefrontal cortex in experience-based decisions (Yechiam and Aharon, 2011; Yechiam and Hochman, 2013).

Other interesting insights emerging from our study were the involvement of primary somatosensory and sensory (superior occipital) cortices in subserving the higher-order process of loss aversion, along with ventral prefrontal cortex. This supports the contention that there is a network of brain regions underlying the interaction of loss aversion with the source of the losses. This is related to recent work reporting the involvement of visual, pain related areas in higher-order cognitive processes (Thomas et al., 2013; Mancini et al., 2014).

The role of ventral prefrontal cortex, along with ventral striatum (Tom et al., 2007), underlying loss aversion, merits closer investigation. Our results suggest that the ventral prefrontal cortex (vPFC) codes loss aversion when an income shock is due to subject's own decisions, whereas the ventral striatum codes loss aversion independently of the source of the wealth shock.

While we have employed real-time fMRI here (for reviews, see Ruiz et al., 2016; Sitaram et al., 2016), direct manipulations of brain activation, using techniques such as tDCS/TMS, to investigate the causal relation of brain activation in specific regions or network to behavior could also be brought to bear. We believe that the next step is to use this approach of direct manipulation to investigate the relationship of the ventral prefrontal cortex and ventral striatum to changes in loss aversion due to wealth shocks.

Our work is related to that of Kuhnen (2015), who found that learning in an investment task is more effective in the domain of gains than in the losses. The effect was greater if the individual was actively investing herself than investing passively. It is clear that the passive and active investment tasks evoke different learning processes. A similar phenomenon appears to be at work in our study, in that whether a wealth shock is endogenous or exogenous leads to different subsequent behavioral responses.

We acknowledge that our design has some limitations. The shocks occurred in only one fixed order, with a negative shock always preceding the positive one, and all subjects experienced the CONTINGENT task before the RANDOM. Another limitation is that our design does not allow us to distinguish the response to a shock from a dependence of behavior on wealth levels. We believe that the fact that a shock to wealth has occurred is a greater influence on decisions than the actual wealth level itself, but a future experiment that varied wealth level without shocks could investigate this.

In conclusion, individuals exhibit a greater increase in loss aversion when their payoffs experience a negative shock from their own actions rather than an exogenous shock. The effect does not appear for positive shocks to wealth. Neurobiological correlates show that the cortico-subcortical brain network consisting of bilateral striatum, right amygdala, and dorsal anterior cingulate cortex underlies task-independent neural loss aversion. The cortical-sensory network comprising the right ventral prefrontal cortex, right primary somatosensory cortex, and right superior occipital cortex underlies neural loss aversion that is specific to a reaction to losses due to one's own decisions i.e., agency. These results add to our understanding of the neural substrates of loss aversion and how they correlate with the process whereby the losses were incurred.

## Author contributions

VP, SR, SL, CN, and RS designed, analyzed and prepared the manuscript. SR, SL, and RS collected the data.

### Conflict of interest statement

The authors declare that the research was conducted in the absence of any commercial or financial relationships that could be construed as a potential conflict of interest.

## Supplementary material

Figure S1 **Neural Loss Aversion Differences from one run to the next in both the CONTINGENT and RANDOM tasks**. The activation across the four runs in both task manipulations is shown for the brain areas listed in Table 1. The brain activity in Ventral Prefrontal Cortex/Rostral Anterior Cingulate Cortex **(a1, a2)**, right Primary Somatosensory Cortex/Postcentral **(b1, b2)** and right Superior Occipital Cortex **(c1, c2)** ROIs is illustrated.
