# Supplementary figures and images for: The Effect of Wealth Shocks on Loss Aversion: Behavior and Neural Correlates
Source: Front Neurosci. 2017 Apr 27;11:237. doi: 10.3389/fnins.2017.00237 (PMC5406753; doi:10.3389/fnins.2017.00237)

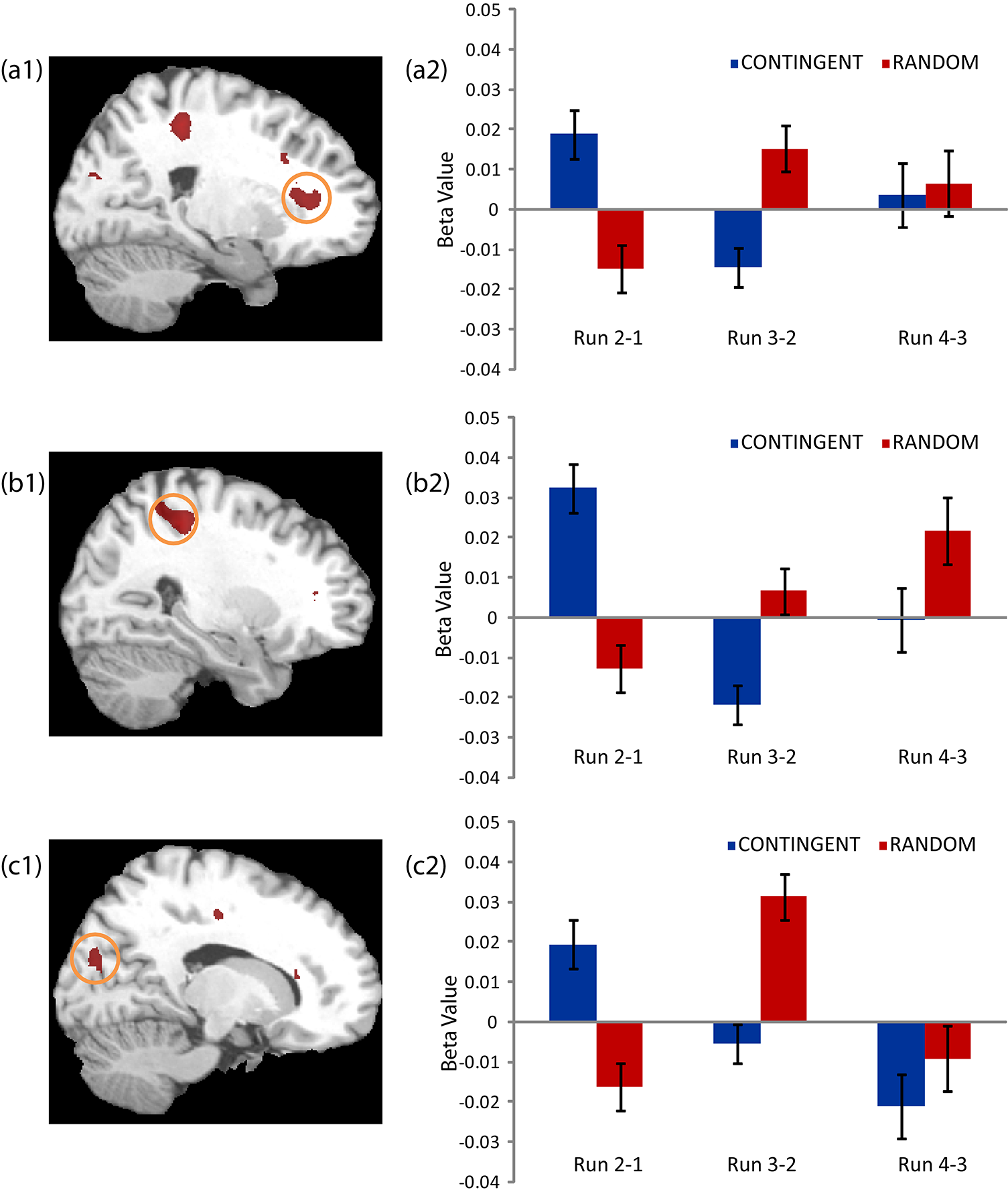

Supplement: Figure S1 — Neural Loss Aversion Differences from one run to the next in both the CONTINGENT and RANDOM tasks. The activation across the four runs in both task manipulations is shown for the brain areas listed in Table 1. The brain activity in Ventral Prefrontal Cortex/Rostral Anterior Cingulate Cortex (a1, a2), right Primary Somatosensory Cortex/Postcentral (b1, b2) and right Superior Occipital Cortex (c1, c2) ROIs is illustrated. [file Image1.TIF]
