# Supplementary material for: The Effect of Wealth Shocks on Loss Aversion: Behavior and Neural Correlates
Source: Front Neurosci. 2017 Apr 27;11:237. doi: 10.3389/fnins.2017.00237 (PMC5406753; doi:10.3389/fnins.2017.00237)
Supplement: Supplementary file 2 [file DataSheet1.PDF]

Contingent: Task Instructions

This experiment allows researchers to investigate how the brain responds to decisions which yield better and worse outcomes. With the initial endowment of 20 Euros given, you will be playing this experimental session consisting of 4 runs of 64 trials each.

Initially you will be asked to choose left-key to ACCEPT the 50/50 gamble and right-key to REJECT the 50/50 gamble. Accepting 50/50 gamble yields a potentially worse or better outcome. Rejecting the gamble yields nothing i.e. you end up with your initial endowment. The occurrences of the gambles have been predetermined by the experimenter so that the odds are really 50/50 and your previous choices do not affect the outcomes on any subsequent trials.

At the end of each run, we will generate your endowment value for the next run. This endowment value will equal the endowment value at the beginning of the previous run plus the outcomes from six random trials in the run that has just finished. Therefore, your final earnings for the session are equal to the initial endowment value of 20 EUR plus the outcomes from 24 randomly chosen trials, six of which are chosen randomly for each run.

The example of a sample trial: In this trial the **accepting** 50/50 gamble yields either 20 units (currency) **loss** or 40 units (currency) **gain**. **Rejecting** the gamble would leave you with nothing and leaves you with the amount that is carried from the previous run. You are allowed to select (accept or reject) *within 3 seconds after the arrow turns to pink*. If you exceed 3 seconds then the program will proceed to the next trial.

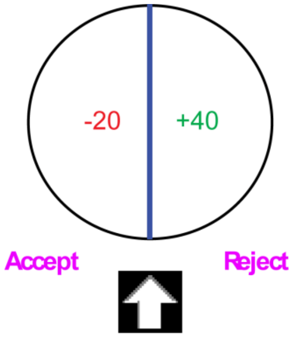

Your gamble option

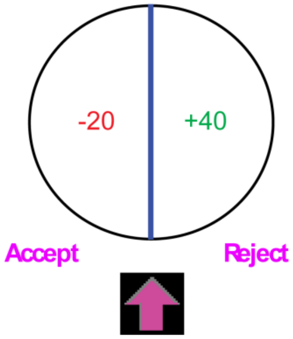

Decide to accept or reject the gamble by moving the pink arrow.

(*Move only when the arrow turns to Pink*)

Decisions chosen in every trial is important and we request you to **put utmost care** in either to accept or reject all the gambles (all 256 trials) in the experiment.

Random: Task Instructions

This experiment allows researchers to investigate how the brain responds to decisions which yield better and worse outcomes. With the fixed endowment for each run, you will be playing this experimental session consisting of 4 runs of 64 trials each.

Initially you will be asked to choose left-key to ACCEPT the 50/50 gamble and right-key to REJECT the 50/50 gamble. Accepting 50/50 gamble yields a potentially worse or better outcome. Rejecting the gamble yields nothing i.e. you end up with your initial endowment. The occurrences of the gambles have been predetermined by the experimenter so that the odds are really 50/50 and your previous choices do not affect the outcomes on any subsequent trials.

At the beginning of each experimental run the endowment might change. This change will not depend on the choices you make. At the end of 4 runs, six trials from each run will be randomly chosen to count toward your final earnings. Your earnings for the session will equal the endowment at the beginning of run 4 plus the outcome of the 24 randomly chosen trials, 6 from each run.

The example of a sample trial: In this trial the **accepting** 50/50 gamble yields either 20 units (currency) **loss** or 40 units (currency) **gain**. **Rejecting** the gamble would leave you with nothing and leaves you with the amount that is fixed for this run. You are allowed to select (accept or reject) *within 3 seconds after the arrow turns to pink*. If you exceed 3 seconds then the program will proceed to the next trial.

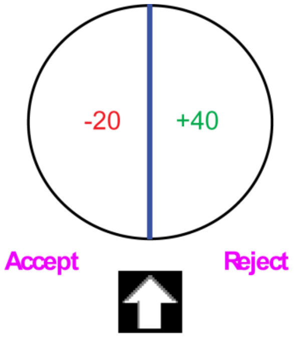

Your gamble option

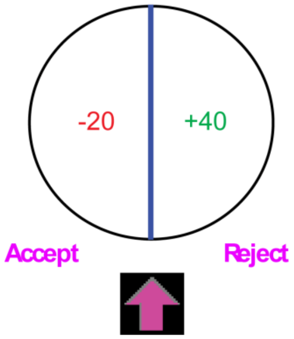

Decide to accept or reject the gamble by moving the pink arrow.

(Move only when the arrow turns to Pink)

Decisions chosen in every trial is important and we request you to **put utmost care** in either to accept or reject all the gambles (all 256 trials) in the experiment.
